# Reply: Correspondence on NanoVar’s performance outlined by Jiang T. et al. in ‘Long-read sequencing settings for efficient structural variation detection based on comprehensive evaluation’

**DOI:** 10.1186/s12859-023-05483-x

**Published:** 2023-09-20

**Authors:** Tao Jiang, Shiqi Liu, Hongzhe Guo

**Affiliations:** Faculty of Computing, Harbin Institute of Technology, Harbin, 150001 China

**Keywords:** NanoVar, Structural variant, Performance benchmarking, Simulated long-read

## Abstract

We published a paper in BMC Bioinformatics comprehensively evaluating the performance of structural variation (SV) calling with long-read SV detection methods based on simulated error-prone long-read data under various sequencing settings. Recently, C.Y.T. et al. wrote a correspondence claiming that the performance of NanoVar was underestimated in our benchmarking and listed some errors in our previous manuscripts. To clarify these matters, we reproduced our previous benchmarking results and carried out a series of parallel experiments on both the newly generated simulated datasets and the ones provided by C.Y.T. et al. The robust benchmark results indicate that NanoVar has unstable performance on simulated data produced from different versions of VISOR, while other tools do not exhibit this phenomenon. Furthermore, the errors proposed by C.Y.T. et al. were due to them using another version of VISOR and Sniffles, which caused many changes in usage and results compared to the versions applied in our previous work. We hope that this commentary proves the validity of our previous publication, clarifies and eliminates the misunderstanding about the commands and results in our benchmarking. Furthermore, we welcome more experts and scholars in the scientific community to pay attention to our research and help us better optimize these valuable works.

## Introduction

Establishing guidance on sequencing coverage, read length, and error rate is a fundamental task to maintain high yields of structural variation (SV) and achieve the lowest cost. We implemented this through comprehensive evaluations of the performance of several state-of-the-art SV calling methods on a full range of simulated error-prone long-read datasets containing various sequencing settings. Recently, C.Y.T. et al., the authors of NanoVar [1], argued that their SV calling performance was underestimated in our benchmarking and proposed some “detrimental errors.“ We confirmed the correctness of our previous benchmarking results. However, to identify more underlying reasons, we set up multiple sets of parallel experiments to explain NanoVar’s poor performance and the “errors” encountered by C.Y.T. et al.

## Benchmark on NanoVar and other controlled SV callers

**Fig. 1:**
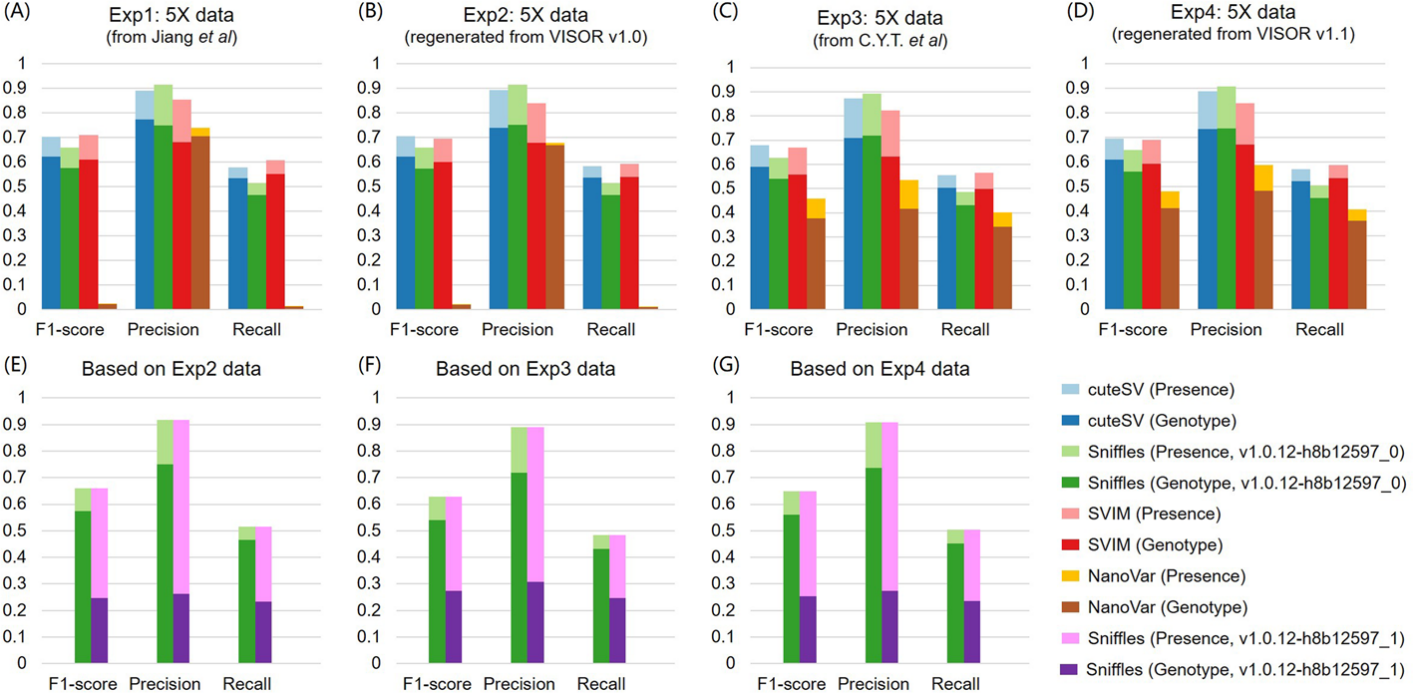
Benchmarking of the SV callers using 5× simulated datasets generated from different versions of VISOR. The F1-scores, precision, and recall for each SV caller were evaluated on four different datasets: **A** from our previous paper, **B** regenerated from VISOR v1.0, **C** from C.Y.T. et al., and **D** regenerated from VISOR v1.1. Additionally, we also evaluated the F1-scores, precision, and recall of two versions of Sniffles v1.0.12 on the data of Exp2 to Exp4, which are presented in (**E** to **G**)

**Fig. 2:**
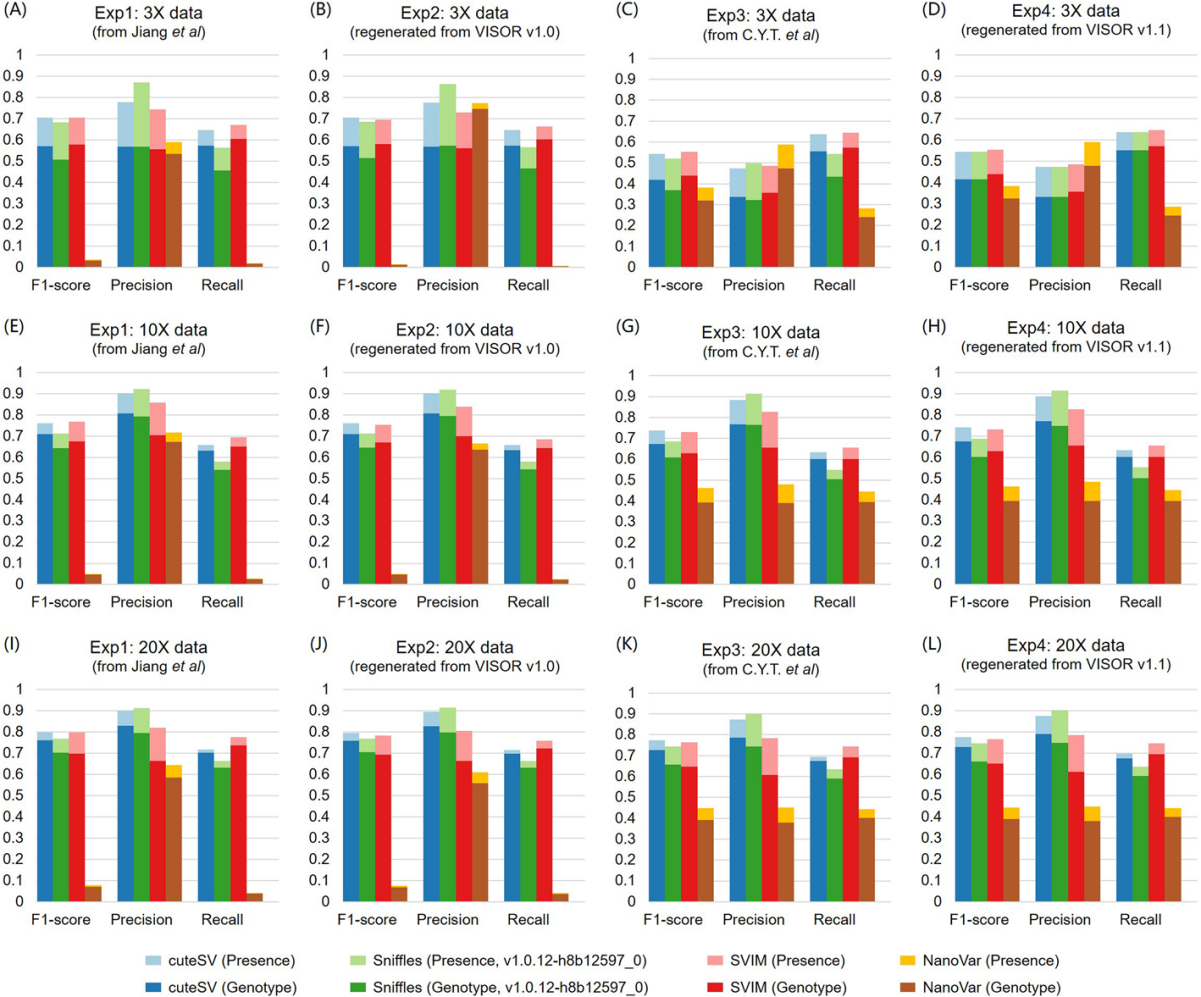
Benchmarking of the SV callers using 3×/10×/20× simulated datasets generated from different versions of VISOR.** A–D** The F1-scores, precision, and recall for each SV caller on four 3× different datasets. **E–H** The F1-scores, precision, and recall for each SV caller on four 10× different datasets. **I–L** The F1-scores, precision, and recall for each SV caller on four 20× different datasets

Here we conducted four sets of parallel experiments using 5× simulated datasets (refer to Table 1) to reevaluate the performance of NanoVar (v1.3.8), and we included Sniffles [2] (1.0.12), SVIM [3] (v1.4.0), and cuteSV [4] (v1.0.10) as controls. Firstly, we presented the performance of each SV caller in our previous paper (Fig. 1A, referred to as Exp1) as a relative standard. To reproduce these results, we generated a new simulated dataset using VISOR [5] (v1.0) with the previously provided commands (https://github.com/SQLiu-youyou/The-commands-of-the-evaluation), and we evaluated the SV calling results using Truvari [6] (version 2.1, which was used in our paper). The assessment results are shown in Fig. 1B (labeled as Exp2), and the performances of the four SV detection tools broadly agree with Exp1. Next, we downloaded the 5× data provided by C.Y.T. et al. from Zenodo (doi:10.5281/zenodo.5856460), and evaluated the four approaches (Fig. 1C, labeled as Exp3). The benchmark results for cuteSV, Sniffles, and SVIM were still consistent with Exp1 and 2, while the performance of NanoVar was different from before. For instance, when considering SV presence assessment, the precision decreased about 20%, whereas the recall and F1 score increased about 39% and 44%, respectively. In this experiment, we found that C.Y.T. et al. applied a new version of VISOR (v1.1) to produce this simulated data, which might be the potential reason why they achieved a higher performance of NanoVar. Meanwhile, this new version of VISOR also caused changes in its usage (e.g., parameters, genomic coordinates and etc., which we explain in the next section). To prove whether the performance change of NanoVar is caused by the different version of VISOR, we regenerated a new simulated dataset through this recently released VISOR. From the results shown in Fig. 1D (labeled as Exp4), it is clear that cuteSV, Sniffles, and SVIM still achieved higher performances consistent with Exp1 to 3, while NanoVar obtained better results compared to Exp1 to 2 and was broadly in agreement with Exp3, which confirmed our conjecture. It is also noted that the same results (Fig. 2) were obtained when repeating the Exp 1 to 4 on the 3×, 10×, and 20× data respectively. To further explore the underlying causes of the performance changes for NanoVar, we manually reviewed the running log files and SV call files produced by NanoVar when employing various versions of VISOR. All log files showed that NanoVar worked regularly and completed properly. However, the number of SV calls under VISOR v1.1 was significantly more than that under v1.0. Hence, it is not so much that we underestimated NanoVar’s performance as that NanoVar may have compatibility issues with the old version of VISOR. Overall, this phenomenon has not appeared in other tools since they could maintain stable and consistent benchmarking performance.

**Table 1:** The detailed description of the four experiments

	Sequencing settings	VISOR version
Exp. 1	Coverage = 5$\times$ Read length = 20 kbp Error rate = 10% Platform = PacBio	v1.0
Exp. 2		v1.0
Exp. 3		v1.1
Exp. 4		v1.1

For the issue of different genotype performance results observed for Sniffles between C.Y.T. et al. and us, it is still due to the different versions of Sniffles that we used. There are actually two versions of Sniffles marked as v1.0.12 available on Bioconda, i.e., sniffles-1.0.12-h8b12597_0, which we used, whereas sniffles-1.0.12-h8b12597_1 was used by C.Y.T. et al. We evaluated the two versions of Sniffles using the data from Exp 2–4, respectively (Fig. 1E–G). It is obvious that both Sniffles achieved the same SV-presence performance for each dataset but significantly different SV-genotype performances. The sniffles-1.0.12-h8b12597_1 used by C.Y.T. et al. was less than half of what we applied. This difference in performance and adaptability is an objective reality, and in our previous paper, we adopted an ensemble SV calling method to improve the variant concordance between every single caller and provide better guidelines for selecting long-read sequencing settings for efficient SV calling.

### Clarification of errors C.Y.T. et al. encountered during the analysis

As mentioned above, we used version 1.0 of VISOR in our previous work, which differed from version 1.1 in three ways, as far as we know: (1) the parameter “-bed” was changed to “-b”; (2) the start coordinates of genomic regions were converted from 0-based (refer to https://github.com/davidebolo1993/VISOR/blob/master/Examples/SHORtS.LASeR.bed) to 1-based; (3) the previous long-read simulator PBSIM [7] was replaced with Badread [8], and the latter generated read names containing symbols like “comma”. These differences in VISOR usage should not be considered as errors in our previously provided commands (https://github.com/SQLiu-youyou/The-commands-of-the-evaluation).

Another issue that needs to be explained is encountered when employing Truvari to evaluate performance. Truvari is a benchmarking tool with stringent VCF format requirements. For callers that do not follow the format exactly, a few post-processing operations are necessary. These include modifying unclearly defined header entries or discarding illegal SV calls to complete benchmarking. For example, SV call sets generated from Sniffles need to: (1) sort SVs by genomic coordinate, (2) manually add a header description of “STRANDBIAS.” Similarly, SV call sets produced by NanoVar need to remove calls containing “SVLEN=.” or “SVLEN=>”. More details on post-processing commands are available at https://github.com/SQLiu-youyou/The-commands-of-the-evaluation.

## Outlook

In this reply, we conducted a series of parallel experiments to replicate benchmarking results in both our publication and the work presented by C.Y.T. et al. We also explored the key cause of the disagreement between us, which was the different versions of VISOR and Sniffles used. Through this reply, we have demonstrated the reproducibility and accuracy of our past research. Moreover, this highlights the need for future SV caller tools to not only test the stability and compatibility of software performance but also generate SV detection files that adhere better to the VCF format.

## Data Availability

Since Exp1 is our previous published work, here we only provide the data from Exp2 to Exp4. The simulated bam files used in Exp2 and Exp4 are available at https://u.pcloud.link/publink/show?code=VZp8YaVZthc7HpxJEjV4O7iEI5jd1YgS0eQk (please download all files and then implement “cat * > toCTY.tar && tar xvf toCTY.tar” to obtain all the data). The simulated bam file that C.Y.T. et al. generated (i.e., Exp3) is deposited on Zenodo with doi:10.5281/zenodo.5856460. Truvari results of each benchmark are deposited on Zenodo with doi:10.5281/zenodo.8253984. The detailly commands used in this manuscript can be available at https://github.com/SQLiu-youyou/The-commands-of-the-evaluation.

## Abbreviations

SV: Structural variation
